# A pooled analysis of the risk prediction models for mortality in acute exacerbation of chronic obstructive pulmonary disease

**DOI:** 10.1111/crj.13606

**Published:** 2023-03-21

**Authors:** Zile Ji, Xuanlin Li, Siyuan Lei, Jiaxin Xu, Yang Xie

**Affiliations:** ^1^ Department of Respiratory Diseases The First Affiliated Hospital of Henan University of Chinese Medicine Zhengzhou China; ^2^ Co‐Construction Collaborative Innovation Center for Chinese Medicine and Respiratory Diseases by Henan & Education Ministry of P.R. China Henan University of Chinese Medicine Zhengzhou China

**Keywords:** AECOPD, mortality, pooled analysis, prediction models

## Abstract

**Objective:**

The prognosis for acute exacerbation of chronic obstructive pulmonary disease (AECOPD) is not optimistic, and severe AECOPD leads to an increased risk of mortality. Prediction models help distinguish between high‐ and low‐risk groups. At present, many prediction models have been established and validated, which need to be systematically reviewed to screen out more suitable models that can be used in the clinic and provide evidence for future research.

**Methods:**

We searched PubMed, EMBASE, Cochrane Library and Web of Science databases for studies on risk models for AECOPD mortality from their inception to 10 April 2022. The risk of bias was assessed using the prediction model risk of bias assessment tool (PROBAST). Stata software (version 16) was used to synthesize the C‐statistics for each model.

**Results:**

A total of 37 studies were included. The development of risk prediction models for mortality in patients with AECOPD was described in 26 articles, in which the most common predictors were age (*n* = 17), dyspnea grade (*n* = 11), altered mental status (*n* = 8), pneumonia (*n* = 6) and blood urea nitrogen (BUN, *n* = 6). The remaining 11 articles only externally validated existing models. All 37 studies were evaluated at a high risk of bias using PROBAST. We performed a meta‐analysis of five models included in 15 studies. DECAF (dyspnoea, eosinopenia, consolidation, acidemia and atrial fibrillation) performed well in predicting in‐hospital death [C‐statistic = 0.91, 95% confidence interval (CI): 0.83, 0.98] and 90‐day death [C‐statistic = 0.76, 95% CI: 0.69, 0.82] and CURB‐65 (confusion, urea, respiratory rate, blood pressure and age) performed well in predicting 30‐day death [C‐statistic = 0.74, 95% CI: 0.70, 0.77].

**Conclusions:**

This study provides information on the characteristics, performance and risk of bias of a risk model for AECOPD mortality. This pooled analysis of the present study suggests that the DECAF performs well in predicting in‐hospital and 90‐day deaths. Yet, external validation in different populations is still needed to prove this performance.

## Graphical abstract

We conducted a meta‐analysis of AECOPD mortality risk prediction models. The results showed that DECAF performs well in predicting in‐hospital death [C‐statistic = 0.91, 95%CI: 0.83–0.98] and 90‐day death [C‐statistic = 0.76, 95%CI: 0.69–0.82] and CURB‐65 performs well in predicting 30‐day death [C‐statistic = 0.74, 95%CI: 0.70–0.77].

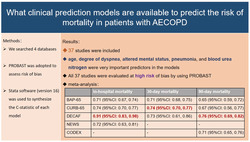

## 1 INTRODUCTION

Chronic obstructive pulmonary disease (COPD) is a slowly progressing incurable respiratory disease that causes morbidity and mortality worldwide. In many countries, the prevalence of COPD has increased steeply with age, with the highest prevalence amongst those aged >60 years.^1^ The Global Burden of Disease Study estimated that the global incidence of COPD was 3.9% in 2017, with 41.9 deaths from COPD per 100 000 people worldwide per year, representing the highest case fatality among chronic respiratory diseases.^2^ Acute exacerbation of COPD (AECOPD) is defined as an acute worsening of respiratory symptoms that necessitate additional therapy.^3^ The economic burden of treatment for COPD exacerbations accounts for the largest proportion of the cost of the disease. Studies have shown that hospitalization for AECOPD is independently associated with mortality.^4^ Mortality after AECOPD ranges from 3.6% of short‐term mortality (within 90 days) to 31% of long‐term mortality (between 90 days and 2 years), and the mortality rate of patients admitted to intensive care units (ICUs) is as high as 29%.^5^ Therefore, early assessment of the prognosis of patients with AECOPD and timely adjustment of treatment options can help reduce mortality and combat negative emotions.

A clinical risk prediction model is a mathematical equation that relates multiple predictive factors to disease diagnosis or prognosis.^6^ As a quantitative tool for risk and benefit assessment, the prediction model can distinguish between low‐risk and high‐risk populations, which is helpful in upgrading the treatment plan or prescribing palliative treatment for a high‐risk population. We have found that mortality from AECOPD is associated with multiple independent predictors, such as age, low body mass index and heart failure, among others.^5^ Notably, the multidimensional scoring system can better predict subsequent survival than a single predictor.^7^ Previous studies have constructed research maps of prognostic models for patients with COPD but have not limited disease stage and prognosis.^8^ Therefore, knowledge on prediction models for mortality in patients with AECOPD is limited. In addition, the efficacy and accuracy of these prognostic models differ; thus, strict review and screening are required for clinical application. This study aimed to systematically review prediction models for the risk of mortality from AECOPD to help clinical decision‐makers select appropriate prediction models.

## 2 METHODS

The protocol for this review was registered in the International Prospective Register of Systematic Reviews (PROSPERO), and the registration number is CRD42022328505.

### 2.1 Search strategy

PubMed, EMBASE, Cochrane Library, and Web of Science databases were searched from their inception to 10 April 2022. The search terms applied were as follows: (‘acute exacerbation of chronic obstructive pulmonary disease’ OR ‘AECOPD’ OR ‘acute exacerbation of COPD’ OR ‘exacerbation of COPD’ OR ‘COPD exacerbation’) AND (‘predict*’ OR ‘progn*’ OR ‘score’ OR ‘risk calculation’ OR ‘risk assessment’ OR ‘risk factor’ OR ‘model’ OR ‘machine learning’ OR ‘artificial intelligence’ OR ‘algorithm’ OR ‘deep learning’ OR ‘regression’) AND (‘death’ OR ‘mortality’ OR ‘survival’). Additionally, we manually searched for references and relevant articles to identify additional studies. The detailed retrieval strategies and steps are presented in Table S1.

### 2.2 Study selection

We included articles written in English that developed or validated prediction models for mortality risk in patients with AECOPD. Meanwhile, studies with incomplete data, duplicate publications, conference abstracts and study protocols were excluded.

### 2.3 Data extraction

Two reviewers (ZLJ and SYL) independently screened the literature and collected data, including author information, year of publication, country, research type, prediction results, sample size, predictors, model discrimination and calibration, modelling method and methods for handling missing data. In cases of disagreement, decisions were made following discussion with a third investigator (YX).

### 2.4 Assessment of risk of bias

The prediction model risk of bias assessment tool (PROBAST)^9^ (Table S2) was used to assess the quality of the included studies, with 20 questions in four key domains: participants, predictors, outcome and analysis. Each question was answered with ‘yes/probably yes’, ‘no/probably no’ and ‘no information’. Moreover, the evaluation results of each domain were judged using ‘low’, ‘high’ or ‘unclear’. Assessments were performed independently by two investigators (ZLJ and JXX), and in cases of disagreement, decisions were made following discussion with a third investigator (YX).

### 2.5 Statistical analysis

A descriptive analysis was used to summarize the general findings of the predictive models, and the frequencies of the variables were calculated. In addition, a random‐effects meta‐analysis using STATA software (version 16) was used to synthesize C‐statistics from multiple studies validating the same model.^10^ Between‐study heterogeneity was quantified using the *I* ^*2*^ statistic. If *I* ^*2*^ was >50%, the studies were considered statistically heterogeneous. The STATA command is listed in Table S3. The meta‐analysis was summarized in a forest plot showing pooled performance.

## 3 RESULTS

### 3.1 Study selection

**FIGURE 1:**
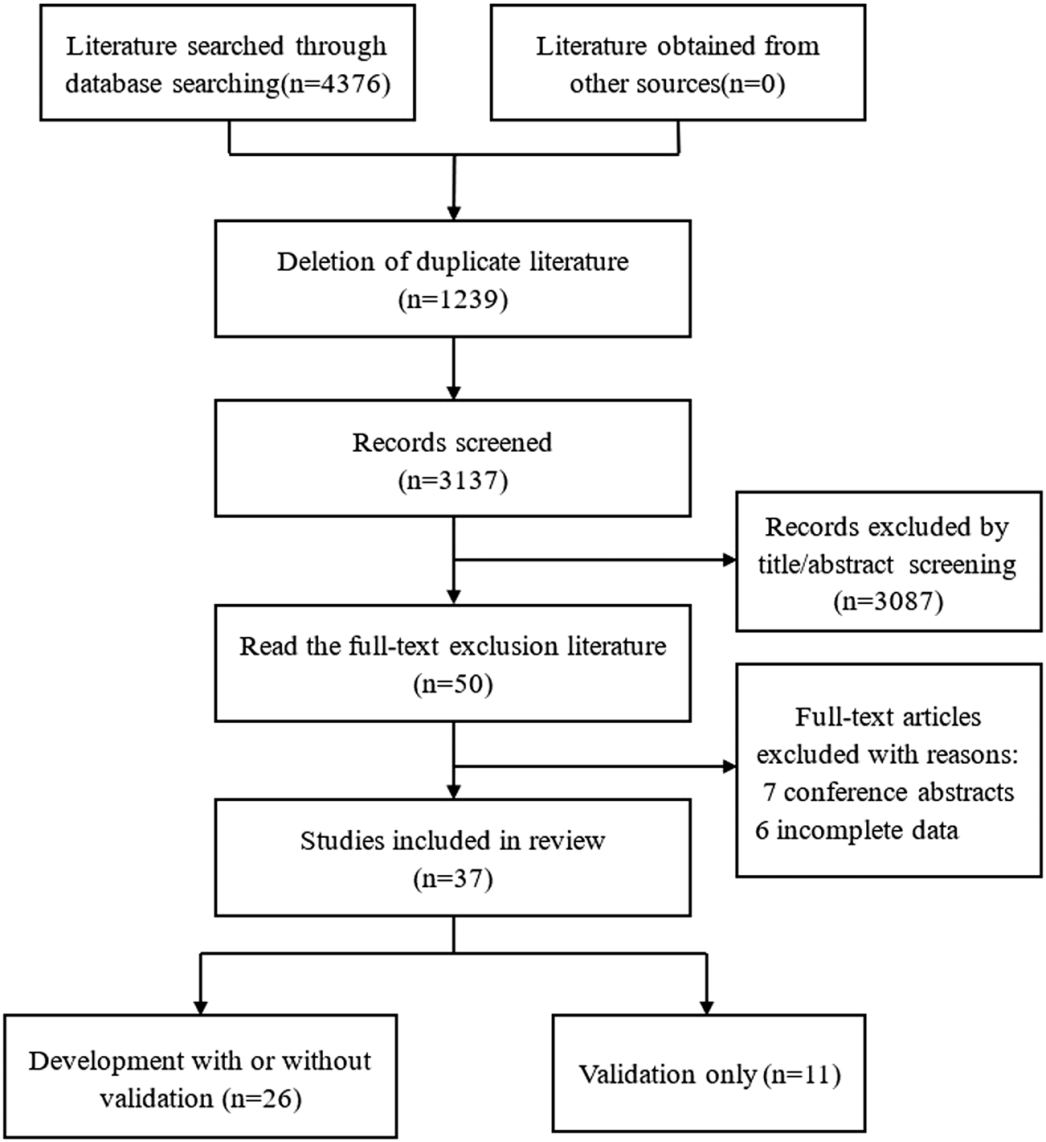
Literature screening flow chart.

A total of 4376 pieces of literature were obtained through database searching, and 37 pieces of literature^11^, ^12^, ^13^, ^14^, ^15^, ^16^, ^17^, ^18^, ^19^, ^20^, ^21^, ^22^, ^23^, ^24^, ^25^, ^26^, ^27^, ^28^, ^29^, ^30^, ^31^, ^32^, ^33^, ^34^, ^35^, ^36^, ^37^, ^38^, ^39^, ^40^, ^41^, ^42^, ^43^, ^44^, ^45^, ^46^, ^47^ were finally included after the screening. The process and results are shown in Figure 1. A list of excluded studies and reasons for exclusion are provided in Table S4. This study included 26 studies^11^, ^12^, ^13^, ^14^, ^15^, ^16^, ^17^, ^18^, ^19^, ^20^, ^21^, ^22^, ^23^, ^24^, ^25^, ^26^, ^27^, ^28^, ^29^, ^30^, ^31^, ^32^, ^33^, ^34^, ^35^, ^36^ that developed models with or without validation and 11 studies^37^, ^38^, ^39^, ^40^, ^41^, ^42^, ^43^, ^44^, ^45^, ^46^, ^47^ that only validated models. Fifteen studies^30^, ^32^, ^34^, ^36^, ^37^, ^38^, ^39^, ^40^, ^41^, ^42^, ^43^, ^44^, ^45^, ^46^, ^47^ were finally included for quantitative statistical analysis.

### 3.2 Characteristics of the 26 studies that developed models with or without validation

**FIGURE 2:**
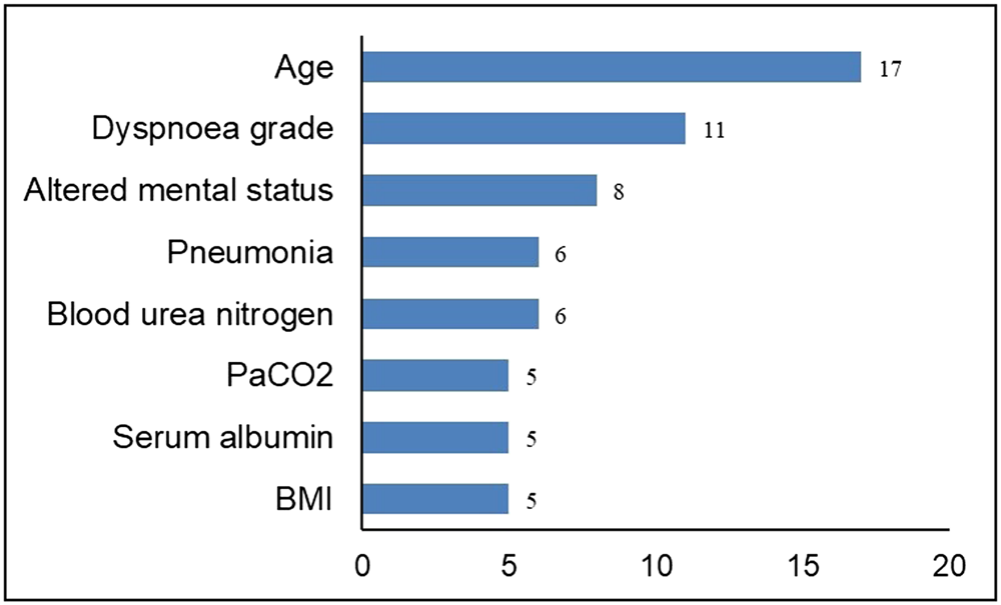
Predictors in 26 risk prediction models for AECOPD mortality. AECOPD, acute exacerbation of chronic obstructive pulmonary disease; BMI, body mass index; PaCO_2_, partial pressure of carbon dioxide.

We found 26 studies^11^, ^12^, ^13^, ^14^, ^15^, ^16^, ^17^, ^18^, ^19^, ^20^, ^21^, ^22^, ^23^, ^24^, ^25^, ^26^, ^27^, ^28^, ^29^, ^30^, ^31^, ^32^, ^33^, ^34^, ^35^, ^36^ describing the development of risk prediction models for mortality in patients with AECOPD. In‐hospital death (*n* = 15) was the most common predictor of mortality. The prediction models were mainly built in the United States (*n* = 5), Spain (*n* = 5) and China (*n* = 3). The sample sizes ranged from 61 to 150 035 for the development cohort and 284 to 149 646 for the validation cohort. The characteristics of the model building are listed in Table 1. Additional details are provided in Tables S5 and S6. The prediction models were built with different situations: six emergency departments, three ICUs, one primary care and 16 studies^13^, ^14^, ^15^, ^16^, ^17^, ^18^, ^19^, ^22^, ^25^, ^26^, ^27^, ^30^, ^33^, ^34^, ^35^, ^36^ did not specifically address a particular hospitalization setting, which we summarized as an inpatient setting. Internal validation of the model was performed using bootstrapping (*n* = 9), random splitting (*n* = 7) and a combination of methods (*n* = 2). The two most frequently used modelling methods were logistic regression (*n* = 20) and classification and regression tree (*n* = 3). Many studies did not report how to handle missing values, and imputation (*n* = 6) was used for the few models for which this was performed. Four studies^18^, ^33^, ^34^, ^35^ assessed the calibration of the model using the Hosmer–Lemeshow test and calibration plot, and the Hosmer–Lemeshow test was the most frequently used calibration method. Many studies adopted the sum score (*n* = 14) to represent the model, and four studies^11^, ^13^, ^31^, ^34^ reported this equation. As shown in Figure 2, among the 26 prediction models, the most commonly used predictors were age (*n* = 17), dyspnoea grade (*n* = 11), altered mental status (*n* = 8), pneumonia (*n* = 6) and blood urea nitrogen (BUN, *n* = 6).

**TABLE 1:** Characteristics of the 26 studies that developed models with or without validation.

	Inpatient setting (*n* = 16)	Emergency department (*n* = 6)	Intensive care unit (*n* = 3)	Primary care (*n* = 1)	Overall (*n* = 26)
Internal validation					
Random splitting	3	3	1	0	7
Bootstrapping	6	1	1	1	9
Cross‐validation	0	0	0	0	0
Combination of methods	1	1	0	0	2
None	6	1	1	0	8
Modelling method					
Logistic regression	11	5	3	1	20
Generalized linear model	1	0	0	0	1
Classification and regression tree	2	1	0	0	3
Machine learning	1	0	0	0	1
More than one method	1	0	0	0	1
Not reported	0	0	0	0	0
Handling of missing data					
Imputation	4	1	0	1	6
No missing values	0	0	0	0	0
Inappropriate handling	2	0	0	0	2
Not reported	10	5	3	0	18
Model discrimination					
C‐statistic	16	6	3	1	26
None	0	0	0	0	0
Model calibration					
Hosmer–Lemeshow test	3	1	1	0	5
Calibration plot	1	0	0	1	2
Hosmer–Lemeshow test and Calibration plot	4	0	0	0	4
Other	4	0	0	0	4
None	4	5	2	0	11
Model presentation					
Sum score	9	4	1	0	14
Decision tree	1	1	0	0	2
Nomogram	1	1	0	0	2
Equation	2	0	1	1	4
More than one method	1	0	0	0	1
None	2	0	1	0	3

### 3.3 Characteristics of the 11 studies that only validated the models

As shown in Table 2, 11 articles^37^, ^38^, ^39^, ^40^, ^41^, ^42^, ^43^, ^44^, ^45^, ^46^, ^47^ were the only externally validated existing models, and the sample size ranged from 100 to 3321. Three studies^39^, ^40^, ^43^ were from the United Kingdom. The application occasion for two studies^40^, ^42^ was the emergency room, and three studies^39^, ^42^, ^43^ dealt with missing data using imputation. All studies used C‐statistics to express discrimination, and the Hosmer–Lemeshow test was the most frequently used calibration method. The primary outcome was in‐hospital mortality. Additional details are provided in Tables S7 and S8.

**TABLE 2:** Characteristics of the 11 studies that only validated the models.

	Inpatient setting (*n* = 8)	Emergency department (*n* = 2)	Intensive care unit (*n* = 1)	Overall (*n* = 11)
Handling of missing data				
Imputation	2	1	0	3
No missing values	0	1	0	1
Inappropriate handling	0	0	0	0
Not reported	6	0	1	7
Model calibration				
Hosmer–Lemeshow test	3	0	0	3
Calibration plot	0	1	0	1
Hosmer–Lemeshow test and Calibration plot	0	1	0	1
Other	2	0	0	2
None	3	0	1	4

### 3.4 Risk of bias assessment

**FIGURE 3:**
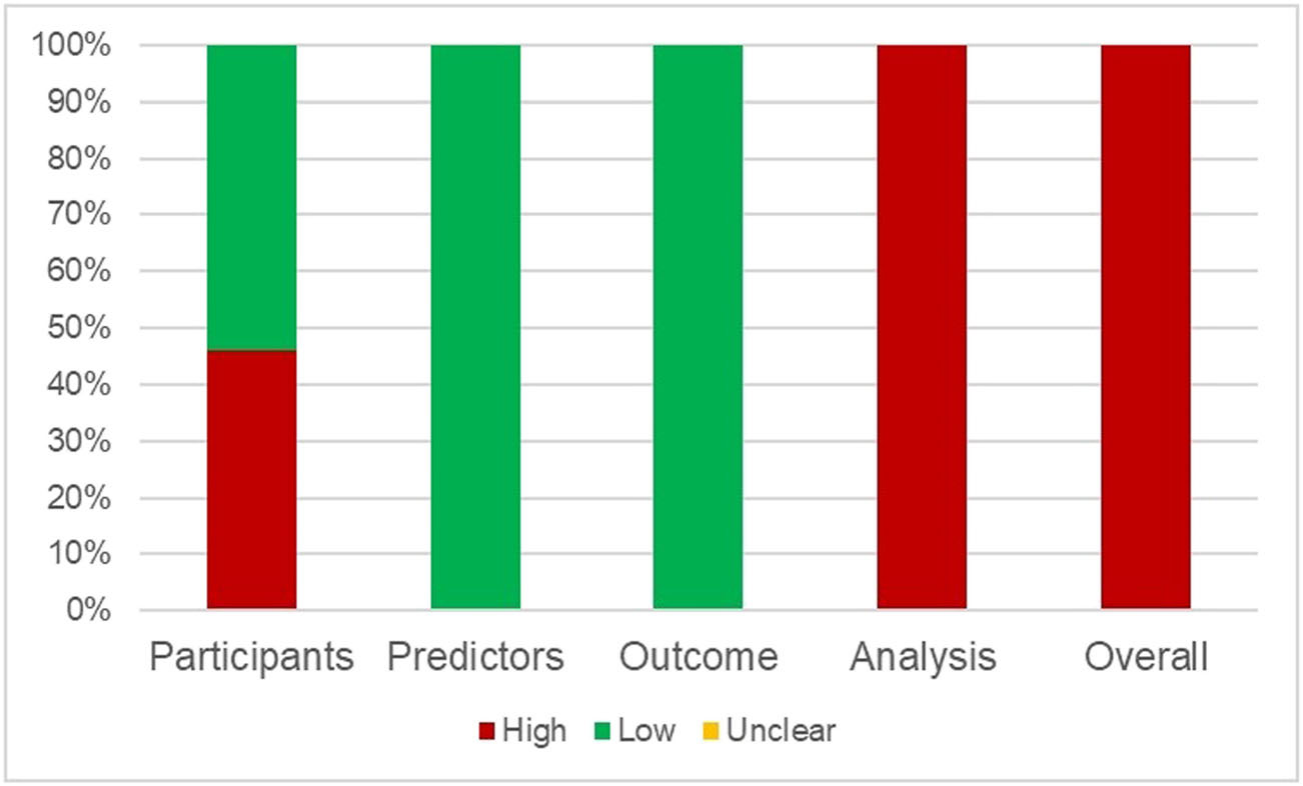
Risk of bias assessment in 37 studies.

We evaluated 37 studies^11^, ^12^, ^13^, ^14^, ^15^, ^16^, ^17^, ^18^, ^19^, ^20^, ^21^, ^22^, ^23^, ^24^, ^25^, ^26^, ^27^, ^28^, ^29^, ^30^, ^31^, ^32^, ^33^, ^34^, ^35^, ^36^, ^37^, ^38^, ^39^, ^40^, ^41^, ^42^, ^43^, ^44^, ^45^, ^46^, ^47^ for the risk of bias using the PROBAST checklist, and all studies were at a high risk of bias, as shown in Figure 3. The main sources of risk were failure to correctly assess predictive model performance (*n* = 29), insufficient sample size (*n* = 27), selection of predictors using univariate analysis (n = 17), inappropriate data sources (*n* = 17), lack of internal validation (*n* = 8), continuous predictors handled inappropriately (*n* = 7) and missing data not handled appropriately (*n* = 2).

### 3.5 Statistical analysis

**FIGURE 4:**
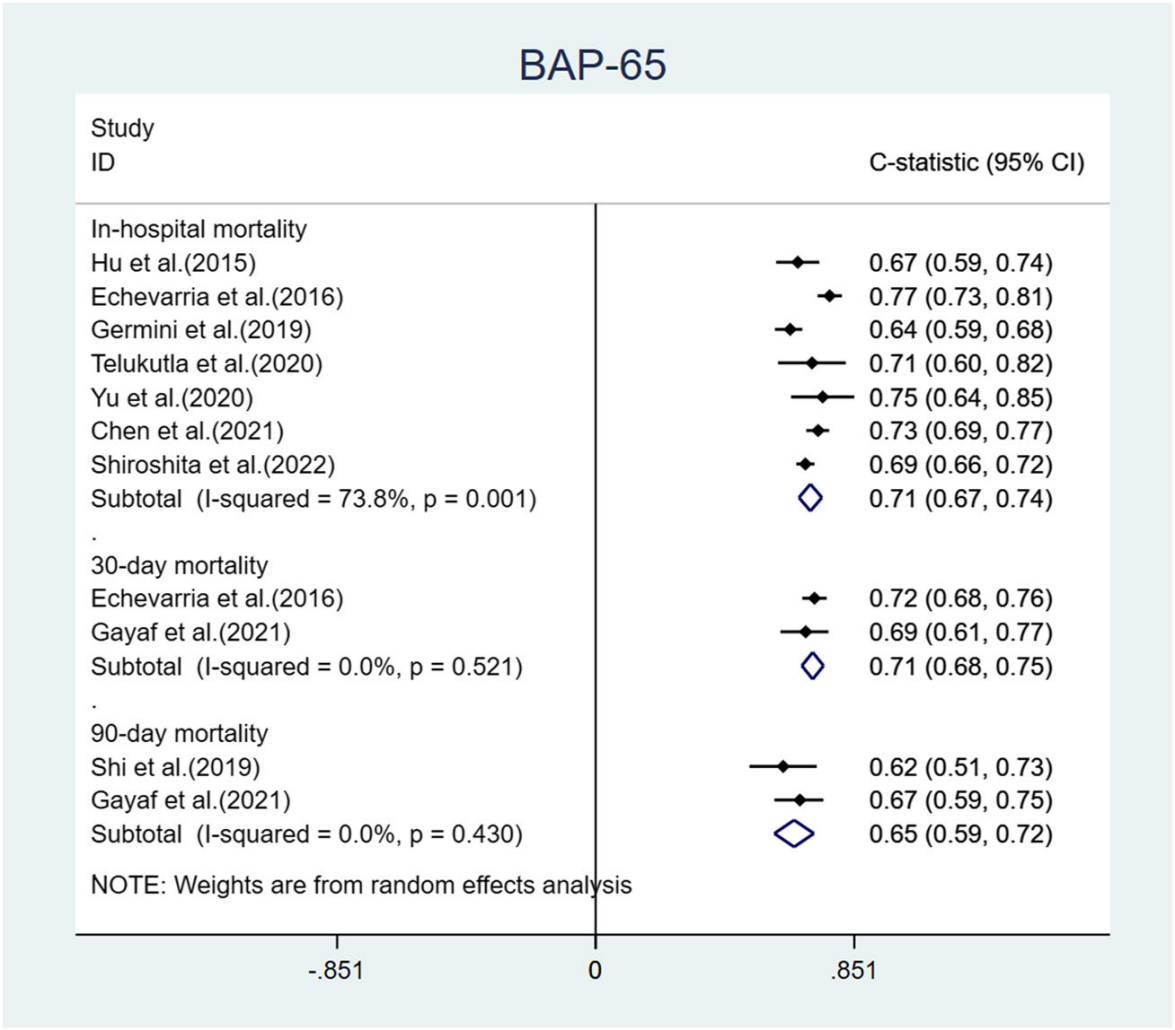
Forest plot showing C‐statistics of BAP‐65 scores in predicting in‐hospital, 30‐day, and 90‐day mortalities.

**FIGURE 5:**
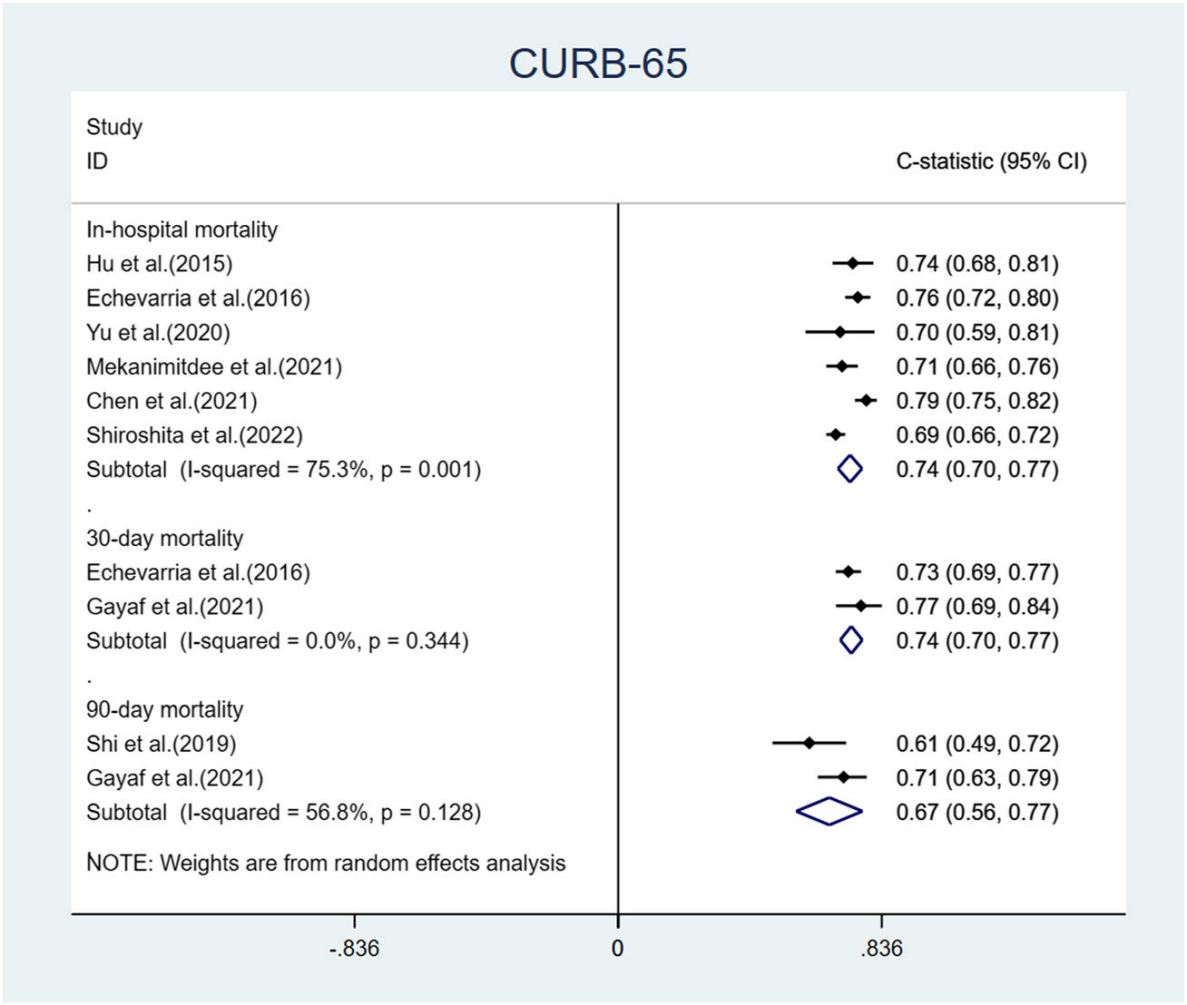
Forest plot showing C‐statistics of CURB‐65 scores in predicting in‐hospital, 30‐day, and 90‐day mortalities.

**FIGURE 6:**
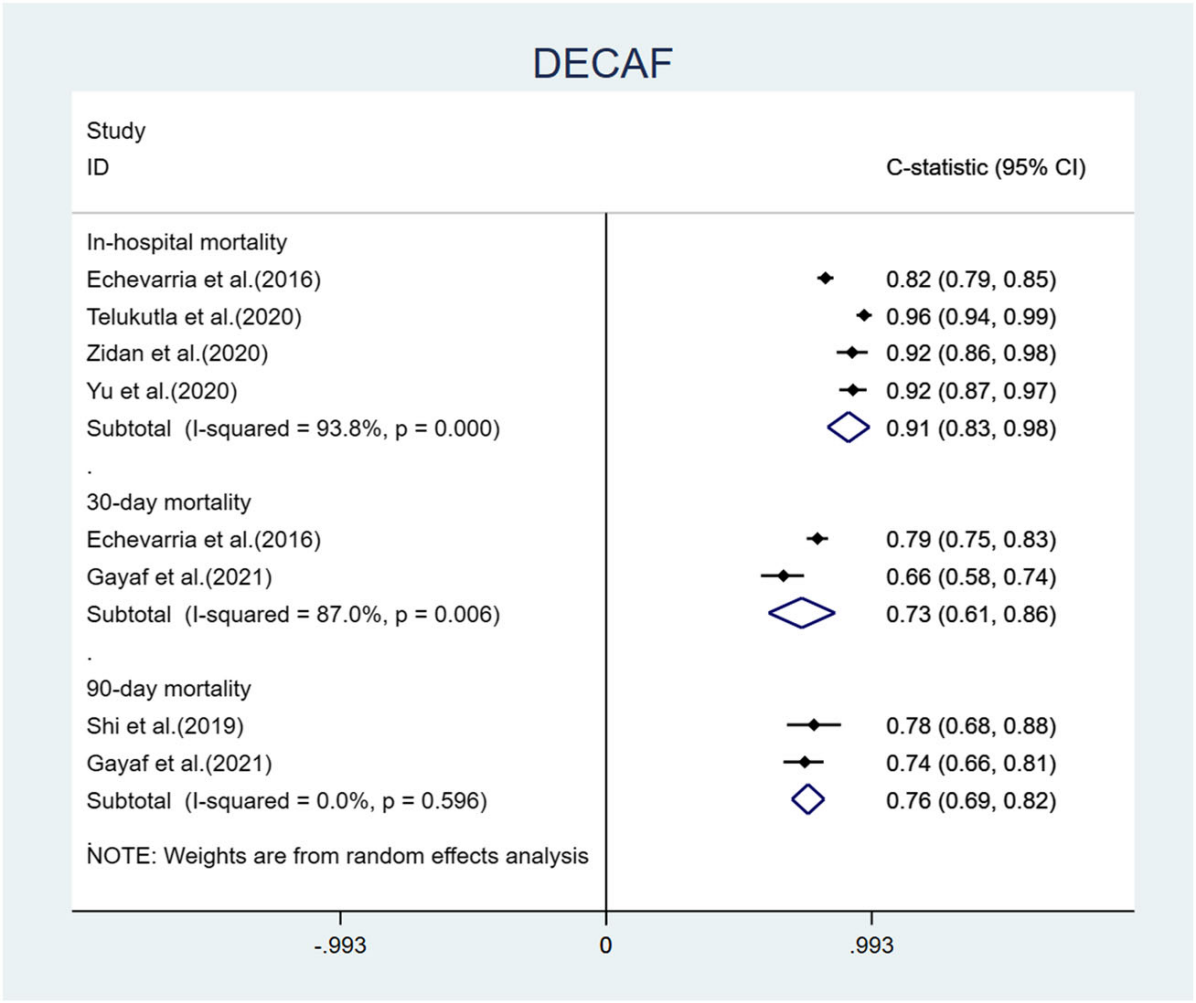
Forest plot showing C‐statistics of DECAF scores in predicting in‐hospital, 30‐day, and 90‐day mortalities.

**FIGURE 7:**
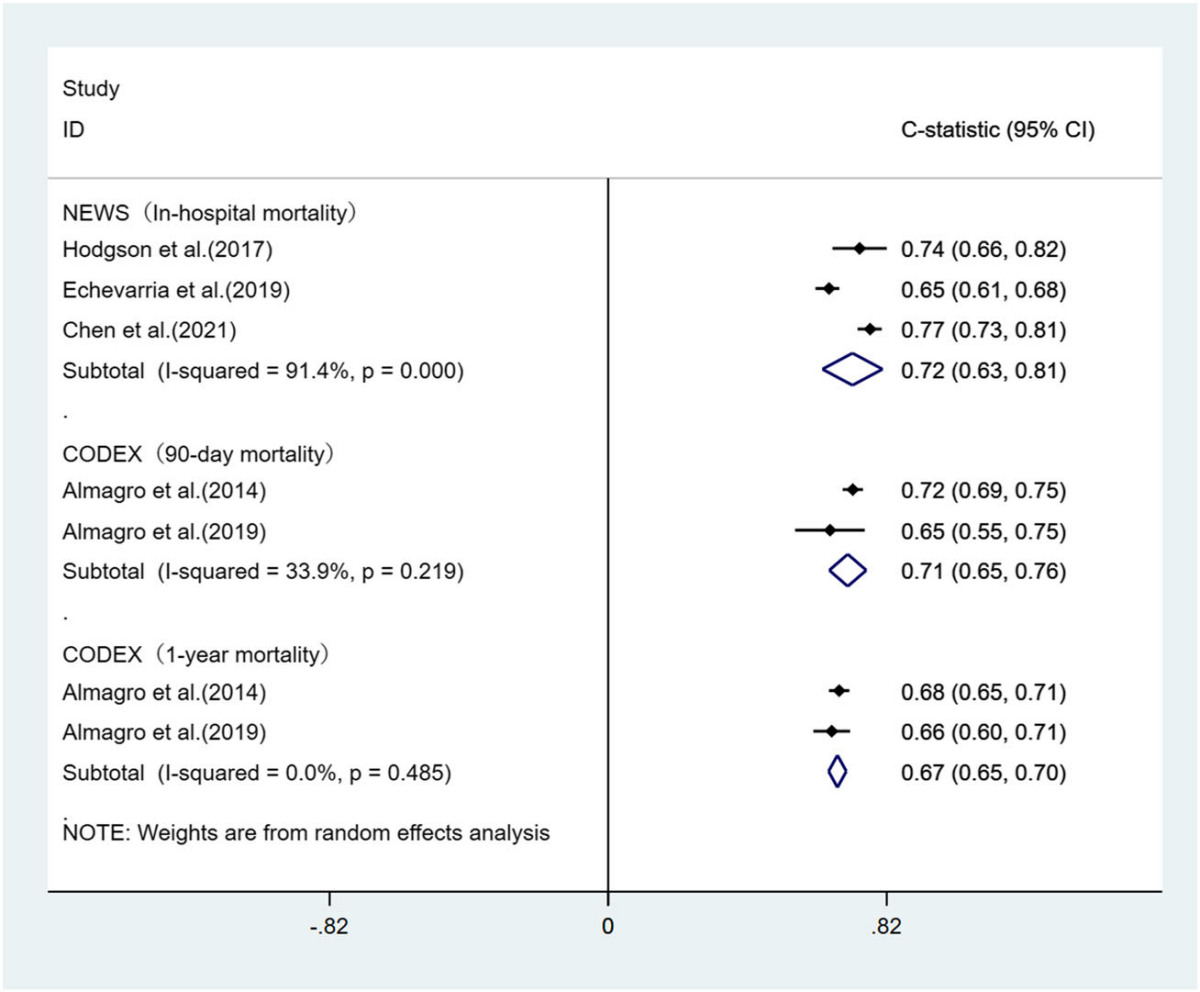
Forest plot showing C‐statistics of NEWS scores in predicting in‐hospital mortality and CODEX scores in predicting 90‐day and 1‐year mortalities.

We performed a meta‐analysis of the C‐statistics of the five models included in the 15 studies.^30^, ^32^, ^34^, ^36^, ^37^, ^38^, ^39^, ^40^, ^41^, ^42^, ^43^, ^44^, ^45^, ^46^, ^47^ More details are provided in Table S9. The results are shown in Figures 4, 5, 6, 7. The pooled C‐statistics of BAP‐65 (BUN, altered mental status, pulse, age), CURB‐65 (confusion, urea, respiratory rate, blood pressure, and age), and DECAF (dyspnoea, eosinopenia, consolidation, acidemia and atrial fibrillation) in predicting in‐hospital mortality were 0.71 (95% confidence interval [CI]: 0.67, 0.74), 0.74 (95% CI: 0.70, 0.77) and 0.91 (95% CI: 0.83, 0.98) respectively. Those for predicting 30‐day mortality were 0.71 (95% CI: 0.68, 0.75), 0.74 (95% CI: 0.70, 0.77) and 0.73 (95% CI: 0.61, 0.86), respectively. Further, those for predicting 90‐day mortality were 0.65 (95% CI: 0.59, 0.72), 0.67 (95% CI: 0.56, 0.77) and 0.76 (95% CI: 0.69, 0.82), respectively. The pooled C‐statistic of NEWS (respiratory rate, oxygen saturation, temperature, systolic blood pressure, pulse rate and level of consciousness) in predicting in‐hospital mortality was 0.72 (95% CI: 0.63, 0.81); the performance of CODEX (comorbidity, obstruction, dyspnoea and previous severe exacerbations) in predicting 90‐day mortality was 0.71 (95% CI: 0.65, 0.76), and that for 1‐year mortality was 0.67 (95% CI: 0.65, 0.70).

## 4 DISCUSSION

This systematic review of risk prediction models for mortality in patients with AECOPD included 37 studies, presenting 26 studies of the model development processes and 11 studies to validate the models. Although a certain number of models can be chosen, there is a high risk of bias for all model developments or validations. Age, dyspnoea grade, altered mental status, pneumonia and urea nitrogen were the most frequently used predictors to develop risk models for mortality from AECOPD. We also performed a meta‐analysis on the external validation of the five models, and the most external validation scales were BAP‐65, CURB‐65 and DECAF.

The present study revealed methodological flaws during model building and validation, which were also reflected in the assessment of the risk of bias. The performance of the model was typically demonstrated by discrimination and calibration, with all studies providing C‐statistics; however, only eight studies^18^, ^26^, ^31^, ^33^, ^34^, ^35^, ^40^, ^42^ provided calibration plots. Calibration assessed by the Hosmer–Lemeshow test has limited applicability for assessing poorer calibration and is sensitive to the number of groups and sample size. The sample size of 27 studies^11^, ^12^, ^15^, ^17^, ^20^, ^22^, ^23^, ^24^, ^25^, ^28^, ^29^, ^30^, ^31^, ^32^, ^33^, ^34^, ^36^, ^37^, ^38^, ^39^, ^40^, ^42^, ^43^, ^44^, ^45^, ^46^, ^47^ was insufficient. Moreover, studies have suggested a minimum of 20 events per independent variable (EPV) for model development,^48^ and an EPV for model validation should be greater than or equal to 100. Seventeen studies^12^, ^15^, ^17^, ^18^, ^20^, ^21^, ^22^, ^23^, ^24^, ^26^, ^27^, ^28^, ^29^, ^30^, ^31^, ^34^, ^35^ employed univariate analysis to screen for predictors that would miss important variables and, therefore, have a high risk of bias. Moreover, there were 17 retrospective cohort studies^14^, ^16^, ^18^, ^19^, ^21^, ^26^, ^28^, ^31^, ^32^, ^33^, ^34^, ^36^, ^37^, ^40^, ^41^, ^42^, ^43^ with a high risk of bias because data from retrospective studies are often inconsistently measured and recorded.^49^ Most studies perform internal validation during model development, which provides more accurate estimates of model performance. The dichotomization of continuous predictors should be avoided because it leads to a loss of information and reduces the model's predictive ability.^50^ Missing data need to be handled appropriately, with only six studies^31^, ^34^, ^36^, ^39^, ^42^, ^43^ in this study dealing with missing values using multiple imputations, which outperformed other methods in controlling bias and precision.^51^ Many studies were completed before the publication of PROBAST. Thus, the assessment of previous studies using the new evaluation criteria may be too stringent.

This study found that age, degree of dyspnoea, altered mental status, pneumonia and BUN were important predictors in the models. Consistent with previous reports,^5^, ^52^ age was a significant predictor of mortality. As age increases, the quality of life and the physical and functional status of various organs continue to decline, resulting in older people being more susceptible to various diseases and a gradually increasing mortality rate. Studies showed that the dyspnoea grade was independently associated with in‐hospital mortality in AECOPD,^53^ and the dyspnoea grade predicted survival more closely than it did according to the percentage of predicted forced expiratory volume in the first second (FEV1).^54^ Most of the included studies used the British Medical Research Council (MRC) scale, modified MRC scale and extended MRC dyspnoea score to assess dyspnoea grade. The altered mental status evaluation was mainly performed using the Glasgow Coma Scale (GCS), which indicates acute cardiopulmonary impairment.^20^ The GCS was first used to evaluate patients with a head injury and is widely used to evaluate patients' mental health.^55^ Studies have shown that the GCS is independently associated with the death of patients with AECOPD in the ICU.^5^ It is estimated that approximately 18% of hospitalized patients with AECOPD have concomitant pneumonia.^56^ Pneumonia is common in patients with AECOPD and is associated with higher mortality.^57^ BUN is a key factor reflecting the intricate interrelationship between patients' nutritional status, protein metabolism and renal status, and high BUN levels can help identify patients with more severe clinical conditions.^58^ The BUN has also been considered an important marker of poor prognosis in respiratory diseases,^59^ and in AECOPD, it may reflect intravascular volume depletion from poor oral intake and hyperventilation in the days before admission.^14^

The DECAF, BAP‐65 and CODEX indices were specifically developed to predict the risk of death from AECOPD. DECAF used the expectation–maximization algorithm for the imputation of missing data and used the bootstrap method for internal validation. Although its EPV is <20 and screening of predictors using univariate analysis causes some bias to the model, the DECAF score has been consistently shown to be a good predictive model because of the simplicity of the measured variables and its external validation in multiple national populations.^60^ The C‐statistic of the DECAF score was 0.91 for in‐hospital deaths and 0.73 for 30‐day deaths in this study, with large heterogeneity that may be related to the fact that the study participants were from different regions and records, where data were inconsistently collected. The BAP‐65 score is also a simpler and more convenient model. Although the model was not assessed for calibration, extensive external validation demonstrated good predictive performance of BAP‐65 with a pooled C‐statistic of 0.71 for both in‐hospital and 30‐day mortalities. The CODEX score is suitable for predicting long‐term mortality in AECOPD (such as 90‐day and 1‐year mortalities); however, its predictive ability is weak. CURB‐65 was originally established to assess the severity of community‐acquired pneumonia.^61^ However, it has also been largely validated in the AECOPD population, with a pooled C‐statistic of 0.74 for both in‐hospital and 30‐day deaths, outperforming BAP‐65. The NEWS is often used to assess the severity of acute diseases to remind clinicians of the deterioration of the disease. It is less commonly used in AECOPD, and additional external validation is needed. Compared with BAP‐65, CURB‐65 and CODEX, DECAF performed best at predicting 90‐day mortality, and further external validation is needed to validate the predictive ability of this model.

One strength of this study was the systematic description of the methodological characteristics during the development and validation of the risk prediction model for AECOPD mortality. A meta‐analysis of the C‐statistics was performed using commonly used external validation models. We also performed a risk of bias assessment of the included studies using the PROBAST. Our limitation is that only English literature was included, and it is possible that high‐quality studies in other languages were missed. Moreover, we did not conduct a meta‐analysis of the calibration of the model because the reports and data were too few to perform that analysis.

## 5 CONCLUSION

This study provides information on the characteristics, performance and risk of bias of a risk model for AECOPD mortality. Despite the development of many models, the number of models that have undergone extensive external validation and can be applied clinically is poor. In addition, the safety, clinical effectiveness and cost‐effectiveness of the models should be considered. The meta‐analysis of the present study suggests that the DECAF performs well in predicting in‐hospital and 90‐day mortalities. However, external validation in different populations is still needed to support this.

## AUTHOR CONTRIBUTIONS

Yang Xie and Xuanlin Li would answer for the design and conception of the article, Zile Ji, Siyuan Lei and Jiaxin Xu would answer for the collection and assembly of materials; Zile Ji, Siyuan Lei and Xuanlin Li would answer for data interpretation and analysis; Zile Ji drafted the manuscript; Xuanlin Li, Siyuan Lei, Jiaxin Xu and Yang Xie revised it. All authors reviewed and approved the final version of the manuscript.

## CONFLICT OF INTEREST STATEMENT

The authors declare that they have no competing interests.

## ETHICS STATEMENT

Not applicable.

## Supporting information


**Table S1:** Search strategy(2022.4.10).
**Table S2:** PROBAST: Assessment of Risk of Bias.
**Table S3:** The STATA command.
**Table S4:** Characteristics of excluded studies.
**Table S5:** Basic characteristics of the 26 studies that developed models (whether validated or not).
**Table S6:** Methodological characteristics of the 26 studies that developed models (whether validated or not).
**Table S8:** Methodological of characteristics of the 11 studies that only validated the models.
**Table S9:** Data from 15 studies used for meta‐analysis

## Data Availability

The datasets used and/or analysed during the current study are available from the corresponding author on reasonable request.
