# Supplementary material for: A pooled analysis of the risk prediction models for mortality in acute exacerbation of chronic obstructive pulmonary disease
Source: Clin Respir J. 2023 Mar 21;17(8):707–18. doi: 10.1111/crj.13606 (PMC10435958; doi:10.1111/crj.13606)
Supplement: Supplementary file 1 — Table S1: Search strategy(2022.4.10). Table S2: PROBAST: Assessment of Risk of Bias. Table S3: The STATA command. Table S4: Characteristics of excluded studies. Table S5: Basic characteristics of the 26 studies that developed models (whether validated or not). Table S6: Methodological characteristics of the 26 studies that developed models (whether validated or not). Table S8: Methodological of characteristics of the 11 studies that only validated the models. Table S9: Data from 15 studies used for meta‐analysis [file CRJ-17-707-s001.docx]

Supplementary Material

Table S1:Search strategy（2022.4.10）

| **Pubmed** | |
| --- | --- |
| Search | Query |
| #1 | (prognosis[MeSH Terms]) OR (progn*[Title/Abstract]) OR (predict*[Title/Abstract]) OR (progn*[Title/Abstract]) OR (score[Title/Abstract]) OR (risk calculation[Title/Abstract]) OR (risk assessment[Title/Abstract]) OR (risk factor[Title/Abstract]) OR (model[Title/Abstract]) OR (machine learning[Title/Abstract]) OR (artificial intelligence[Title/Abstract]) OR (algorithm[Title/Abstract]) OR (deep learning[Title/Abstract]) OR (regression[Title/Abstract]) |
| #2 | “acute exacerbation of chronic obstructive pulmonary disease” [Title/Abstract] OR “AECOPD” [Title/Abstract] OR “acute exacerbation of COPD” [Title/Abstract] OR “exacerbation of COPD” [Title/Abstract] OR “COPD exacerbation” [Title/Abstract] |
| #3 | “mortality” [MeSH Terms] OR“death” [Title/Abstract] OR “mortality” [Title/Abstract] OR “survival” [Title/Abstract] |
| #4 | #1 AND #2 AND #3 |
| **Web of science** | |
| Search | Query |
| #1 | “predict*”[Topic] OR “progn*”[Topic] OR “score”[Topic] OR “risk calculation”[Topic] OR “risk assessment”[Topic] OR “risk factor”[Topic] OR “model”[Topic] OR “machine learning”[Topic] OR “artificial intelligence”[Topic] OR “algorithm”[Topic] OR “deep learning”[Topic] OR “regression”[Topic] |
| #2 | “acute exacerbation of chronic obstructive pulmonary disease” [Topic] OR “AECOPD” [Topic] OR “acute exacerbation of COPD” [Topic] OR “exacerbation of COPD” [Topic] OR “COPD exacerbation” [Topic] |
| #3 | “death” [Topic] OR “mortality” [Topic] OR “survival” [Topic] |
| #4 | #1 AND #2 AND #3 |
| **Embase** | |
| Search | Query |
| #1 | (prognosis)/exp OR (progn*): ab,ti OR (predict*):ab,ti OR (progn*): ab,ti OR (score): ab,ti OR (risk calculation) : ab,ti OR (risk assessment) : ab,ti OR (risk factor) : ab,ti OR (model) : ab,ti OR (machine learning) : ab,ti OR (artificial intelligence) : ab,ti OR (algorithm) : ab,ti OR (deep learning) : ab,ti OR (regression) : ab,ti |
| #2 | (acute exacerbation of chronic obstructive pulmonary disease) /exp OR (acute exacerbation of chronic obstructive pulmonary disease): ab,ti OR (AECOPD) : ab,ti OR (acute exacerbation of COPD) : ab,ti OR (exacerbation of COPD) : ab,ti OR (COPD exacerbation) : ab,ti |
| #3 | (mortality)/exp OR (death) : ab,ti OR (mortality) : ab,ti OR (survival) : ab,ti |
| #4 | #1 AND #2 AND #3 |
| **Cochrane Library** | |
| Search | Query |
| #1 | MeSH descriptor: [prognosis] explode all trees |
| #2 | (predict*):ti,ab,kw OR (progn*):ti,ab,kw OR (score) :ti,ab,kw OR (risk calculation) :ti,ab,kw OR (risk assessment) :ti,ab,kw OR (risk factor) :ti,ab,kw OR (model) :ti,ab,kw OR (machine learning) :ti,ab,kw OR (artificial intelligence) :ti,ab,kw OR (algorithm) :ti,ab,kw OR (deep learning) :ti,ab,kw OR (regression) :ti,ab,kw |
| #3 | #1 OR #2 |
| #4 | (acute exacerbation of chronic obstructive pulmonary disease) :ti,ab,kw OR (AECOPD) :ti,ab,kw OR (acute exacerbation of COPD) :ti,ab,kw OR (exacerbation of COPD) :ti,ab,kw OR (COPD exacerbation) :ti,ab,kw |
| #5 | MeSH descriptor: [mortality] explode all trees |
| #6 | (death) :ti,ab,kw OR (mortality) :ti,ab,kw OR (survival) :ti,ab,kw |
| #7 | #5 OR #6 |
| #8 | #3 AND #4AND #7 |

**Table S2:PROBAST: Assessment of Risk of Bias**

| 1. Participants | 2. Predictors | 3. Outcome | 4. Analysis |
| --- | --- | --- | --- |
| 1.1. Were appropriate data sources used, e.g., cohort, RCT, or nested case–control study data? | 2.1. Were predictors defined and assessed in a similar way for all participants? | 3.1. Was the outcome determined appropriately? | 4.1. Were there a reasonable number of participants with the outcome? |
| 1.2. Were all inclusions and exclusions of participants appropriate? | 2.2. Were predictor assessments made without knowledge of outcome data? | 3.2. Was a prespecified or standard outcome definition used? | 4.2. Were continuous and categorical predictors handled appropriately? |
| - | 2.3. Are all predictors available at the time the model is intended to be used? | 3.3. Were predictors excluded from the outcome definition? | 4.3. Were all enrolled participants included in the analysis? |
| - | - | 3.4. Was the outcome defined and determined in a similar way for all participants? | 4.4. Were participants with missing data handled appropriately? |
| - | - | 3.5. Was the outcome determined without knowledge of predictor information? | 4.5. Was selection of predictors based on univariable analysis avoided?* |
| - | - | 3.6. Was the time interval between predictor assessment and outcome determination appropriate? | 4.6. Were complexities in the data (e.g., censoring, competing risks, sampling of control participants) accounted for appropriately? |
| - | - | - | 4.7. Were relevant model performance measures evaluated appropriately? |
| - | - | - | 4.8. Were model overfitting, underfitting, and optimism in model performance accounted for?* |
| - | - | - | 4.9. Do predictors and their assigned weights in the final model correspond to the results from the reported multivariable analysis?* |

*Development studies only.

**Table S3:** **The STATA command**

| “metan cstatistic lci uci, label(namevar=study) random effect(C-statistic) nowt.” |
| --- |

Table S4:**Characteristics of excluded studies**

| **Author, Year** | **Tittle** | **Reasons** |
| --- | --- | --- |
| Cohen.2014 | Mortality after COPD exacerbation: A risk calculator with notes on utilization of palliative care | Conference abstracts |
| Hansen,.2012 | A dynamic cohort model of chronic obstructive pulmonary disease and its treatments |  |
| Islem.2018 | A model to predict short-term prognosis of acute exacerbation of COPD patients admitted to a Tunisian ICU |  |
| Johansen. B.2012 | Prediction of non-invasive ventilation, mechanical ventilation and mortality among patients with acute exacerbations of chronic obstructive pulmonary disease: A Danish population-based cohort study |  |
| Lim.2020 | Predicting 6-month mortality in COPD: Assessment of prognostic variables to predict COPD mortality |  |
| Sprooten.2017 | CAB score predicts short-term mortality at hospital admission for acute exacerbations of COPD |  |
| Steer.2012 | Predicting mortality in patients hospitalised with acute exacerbations of COPD (AECOPD) requiring assisted ventilation |  |
| **Author, Year** | **Tittle** | **Reasons** |
| Saima Akhter2019 | Assessment and comparison of APACHE II (Acute Physiology and Chronic Health Evaluation), SOFA (Sequential Organ Failure Assessment) score and CURB 65(Confusion; Urea; Respiratory Rate; Blood Pressure), for prediction of inpatient mortality in Acute Exacerbation of Chronic Obstructive Pulmonary Disease | incomplete data |
| chang2010 | Predicting early mortality in acute exacerbation of chronic  obstructive pulmonary disease using the CURB65 score |  |
| edwards2011 | The value of the CRB65 score to predict mortality in exacerbations of COPD requiring hospital admission |  |
| Memon2019 | Role of the DECAF Score in Predicting Inhospital Mortality in Acute Exacerbation of Chronic Obstructive Pulmonary Disease |  |
| nafae2015 | Value of the DECAF score in predicting hospital mortality in patients with acute exacerbation of chronic obstructive pulmonary disease admitted to Zagazig University Hospitals, Egypt |  |
| Sharma2020 | A Study of Modified DECAF Score in Predicting Hospital Outcomes in Patients of Acute Exacerbation of Chronic Obstructive Pulmonary Disease at SMS Medical College, Jaipur |  |

Table S5: Basic characteristics of the 26 studies that developed models (whether validated or not)

| **Study** | **Country** | **Follow-up** | **Study type** | **Age(A)** | **Age(B)** | **Sample size(A)** | **Sample size(B)** | **Subjects with outcome(A)** | **Subjects with outcome(B)** | **Outcome** |
| --- | --- | --- | --- | --- | --- | --- | --- | --- | --- | --- |
| Khilnani et al.(2004) | India | 2002 | p | 60±10 | / | 82 | / | 30 | / | In-hospital mortality |
| Roche et al.(2008) | France | 2003-2004 | p | 72.5±11.8 | | 353 | 334 | total 59 | | In-hospital mortality |
| Mohan et al.(2008) | India | 2004-2006 | p | 60.5±11.4 | / | 151 |  | 38 | / | In-hospital mortality |
| Tabak et al.(2009) | United States | 2004-2006 | r | median (interquartile range), 72(63-79) | median (interquartile range), 72(63-79) | 43893 | 44181 | 774 | 837 | In-hospital mortality |
| Tsimogianni et al.(2009) | Greece | 2001-2004 | p | 68±9 | / | 61 | / | 25 | / | Three-year mortality |
| Asiimwe et al.(2011) | UK | 7 years | r | 71.3±11.7 | 71.4±11.3 | 4986 | 4929 | 452 | 469 | in-hospital mortality |
| Steer et al.(2012) | UK | 2008-2010 | p | 71.3±10 | / | 920 |  | 96 | / | in-hospital mortality and 30-day mortality |
| Tabak et al.(2013) | United States | 2005-2007 | r | 70.7±11.7 | 70.4±11.9 | 69299 | 33327 | 2240 | 957 | in-hospital mortality |
| Lindenauer et al.(2013) | United States | 2007-2009 | r | ≥65 | | 150035 | 149646 | 12933 | 12929 | 30-day mortality |
| Quintana et al.(2014) | Spain | 2008-2010 | p | 72.32±9.81 | 73.23±9.51 | 1243 | 1244 | Short-term mortality:30;1-month mortality:40 | Short-term mortality:29;1-month mortality:48 | Short-term mortality;1-month mortality |
| Batzlaff et al.(2014) | United States | 1995-2009 | r | 70±11 | / | 591 | / | 295 | / | 1-year mortality |
| Roche et al.(2014) | France | 2006-2007 | p | 70.3 ± 11.3 | | 912 | 912 | 23 | 22 | in-hospital mortality |
| Zidan et al.(2015) | Egypt | / | p | 46.46 ± 12.27 | / | 100 |  | 11 | / | in-hospital mortality |
| Esteban et al.(2015) | Spain | 2008-2010 | p | 30-day: death:77.7±8.6；survival:72.5±9.8;30-day: death:77.3±8.9；survival:72.3±9.8 | | 1252 | 1235 | 30-day mortality:46;60-day mortality:79 | 30-day mortality:42 and 60-day mortality:76 | 30-day mortality and 60-day mortality |
| Duenk et al.(2017) | the Netherlands | 2014 | p | 67.5±9.6 | / | 155 | / | 30 | / | 1-year mortality |
| Sakamoto et al.(2017) | Japan | 2010-2013 | r | / | / | 3064 | / | 209 | / | in-hospital mortality |
| Esteban et al.(2018) | Spain | 2008 and 2011 | p | 73.4±10 | 72.7±10.7 | 1420 | 3949 | 369 | 492 | 1-year mortality |
| Jain et al.(2018) | United States | 2001-2012 | r | / | / | 662 | 284 | Total 156 | | death during the ICU admission |
| Arostegui et al.(2019) | Spain | 2008-2010 | p | / | / | 2487 | / | 59 | / | in-hospital mortality |
| Yu et al.(2020) | China | 2015-2017 | p | 77±9.5 | / | 695 | 490、500 | 42 | 51 | hospitalization death |
| Alameda et al.(2021) | Spain | 2013-2014 | r | median (interquartile range),78（69-83） | / | 1696 | / | 17 | / | mortality from any cause 30 days after the last primary care visit |
| Chen et al.(2021) | China | 2018-2020 | r | death:77.9±11.6；survival:76.2±10.2 | / | 601 | / | 19 | / | hospital mortality |
| Dong et al.(2021) | China | 2015-2019 | r | 73±10 | 72±10 | 1096 | 700 | 29 | 12 | in-hospital mortality |
| Mekanimitdee et al.(2021) | Thailand | 2015-2017 | r | death:76.8±11；survival:74.1±11.1 | / | 923 | / | 101 | / | in-hospital mortality |
| Hartley et al.(2021) | UK | 2008-2013;2016-2018 | p and r | 72.8±10.0 | 70.5±9.3 | 489 | 733 | 124 | 147 | in-hospital mortality |
| Shiroshita et al.(2022) | Japan | 2008-2020 | r | 77±8 | | 833 | 357 | 88 | | in-hospital mortality |

Table S6: Methodological characteristics of the 26 studies that developed models (whether validated or not)

| **Study** | **Modeling method** | **Validation** | **Internal validation method** | **AUC(A)** | **Internal validation AUC** | **External validation AUC(B)** | **Calibration** | **methods for handling missing data** |
| --- | --- | --- | --- | --- | --- | --- | --- | --- |
| Inpatient setting | | | | | | | | |
| Roche et al.(2008) | logistic regression | Internal | Random split | 0.79 | / | 0.83 | / | / |
| Mohan et al.(2008) | logistic regression | / | / | 0.73 | / | / | sensitivity , specificity, positive and negative predictive values | / |
| Tabak et al.(2009) | classification and regression tree | Internal and External | bootstrap | 0.72 | / | 0.71 | / | / |
| Tsimogianni et al.(2009) | logistic regression and Cox regression | / | / | 0.83 | / |  | / | / |
| Asiimwe et al.(2011) | Classification and Regression Tree | Internal | Random split | 0.734 | 0.701 | / | sensitivity and specificity | / |
| Steer et al.(2012) | logistic regression | Internal | bootstrap | in-hospital:0.86; | in-hospital:0.86;30 days:0.82 |  | Hosmere-Lemeshow | imputed using the expectation maximisation algorithm |
| Tabak et al.(2013) | logistic regression | External | / | 0.83 | / | 0.84 | Hosmere-Lemeshow、calibration plot | A category for patients with missing laboratory data was created and the mortality of this group was compared and pooled into a reference group |
| Lindenauer et al.(2013) | logistic regression | Internal | Random split | 0.72 | / | 0.723 | / | / |
| Roche et al.(2014) | logistic regression | Internal | Random split | 0.79 | 0.78 |  | sensitivity and specificity | / |
| Duenk et al.(2017) | logistic regression | Internal | bootstrap | 0.87 | / | 0.82 | sensitivity and specificity | Missing data were handled using complete case analysis |
| Sakamoto et al.(2017) | logistic regression | Internal | bootstrap | 0.775 | / | / | calibration plot | / |
| Esteban et al.(2018) | generalized linear mixed models | External | / | 0.747 | / | 0.763 | Hosmere-Lemeshow | / |
| Yu et al.(2020) | logistic regression | External | / | 0.929 | 0.937 | 0.914 | Hosmere-Lemeshow | / |
| Dong et al.(2021) | logistic regression | Internal and External | bootstrap | 0.9147 | 0.90887 | 0.8173 | Hosmere-Lemeshow、.calibration plot、 | / |
| Mekanimitdee et al.(2021) | logistic regression | Internal | bootstrap | 0.82 | / | 0.81 | Hosmere-Lemeshow、 calibration plot slope | multiple imputation |
| Hartley et al.(2021) | logistic regression | External | / | / | / | 0.79 | Hosmere-Lemeshow、calibration plot | imputed using the expectation maximisation algorithm |
| Shiroshita et al.(2022) | machine learning | Internal | Random split、Four-fold cross-validation | 0.76 | 0.72 | / | / | multiple imputation |
| ICU | | | | | | | | |
| Khilnani et al.(2004) | logistic regression | / | / | 0.912 | / | / | / | / |
| Batzlaff et al.(2014) | logistic regression | Internal | bootstrap | 0.68 | 0.687 | / | Hosmere-Lemeshow | / |
| Jain et al.(2018) | logistic regression | Internal | Random split | 0.778 | / | / | / | / |
|  |  |  |  |  |  |  |  |  |
| Emergency department | | | | | | | | |
| Roche et al.(2008) | logistic regression | Internal | Random split | 0.79 | / | 0.83 | / | / |
| Quintana et al.(2014) | logistic regression | Internal | Random split | Short-term mortality:0.85;1-month mortality:0.85 | Short-term mortality:0.88; | / | Hosmere-Lemeshow | missing values were imputed |
| Esteban et al.(2015) | Classification and Regression Tree | Internal | Random split、bootstrap | 30-day mortality:0.835;60-day mortality:0.817 | / | 30-day mortality:0.794;60-day mortality:0.770 | / | / |
| Zidan et al.(2015) | logistic regression | / | / | 0.874 | / | / | / | / |
| Arostegui et al.(2019) | logistic regression | Internal | Random split | / | / | / | / | / |
| Chen et al.(2021) | logistic regression | Internal | bootstrap | 0.94 | / | 0.933 | / | / |
|  |  |  |  |  |  |  |  |  |
| Primary care | | | | | | | | |
| Alameda et al.(2021) | logistic regression | Internal | bootstrap | 0.792 | / | / | calibration plot | multiple imputation |

Table S7: Basic characteristics of the 11 studies that only validated the models

| **Study** | **Country** | **Follow-up** | **Study type** | **Age(A)** | **Sample size(A)** | **Subjects with outcome(A)** | **Outcome** |
| --- | --- | --- | --- | --- | --- | --- | --- |
| Inpatient setting | | | | | | | |
| Almagro et al.(2014) | Spain | 2009-2010 | R | 72.6±9.9 | 606 | 3-month:13;1-year:ni | 3-month and 1-year mortality |
| Hu et al.(2015) | China | 2010-2014 | P | 77.85±8.91 | 752 | 73 | In-hospital mortality |
| Echevarria et al.(2016) | UK | 2012-2014 | P | / | 845 | NI | In-hospital mortality and 30-day mortality |
| Echevarria et al.(2019) | UK |  | R | 73.1±10.2 | 2645 | 228 | In-hospital mortality |
| Almagro et al.(2019) | 8 countries | 2008-2014 | R | 66.9 ± 10.5 | 1175 | NI | mortality for short- |
| Gayaf et al.(2021) | Turkey | 2018-2019 | P | / | 141 | In-hospital mortality:29,30-day:25,90-day:39 | In-hospital,30-day,90-day mortality |
| Telukutla et al.(2020) | India | 2018-2019 | p | survival:59.28±12.11;  death:69.1±12.9 | 170 | 23 | In-hospital mortality |
| Zidan et al.(2020) | Egypt | / | P | 57.46±13.12 | 100 | 9 | In-hospital mortality |
| Emergency department | | | | | | | |
| Hodgson et al.(2017) | UK | 2012-2014 | R | 74 (67–82) | 942 | 42 | In-hospital mortality |
| Germini et al.(2019) | Italy | 2004 | R | 76.2 ±10.7 | 2908 | 110 | In-hospital mortality |
| ICU | | | | | | | |
| Shi et al.(2019) | China | 2016-2017 | p | 77.57 ± 7.22 | 112 | 38 | 90-day mortality |

Table S8: Methodological of characteristics of the 11 studies that only validated the models

| **Study** | **Calibration** | **methods for handling missing data** | **Validated model** |
| --- | --- | --- | --- |
| Inpatient setting | | |  |
| Almagro et al.(2014) | NI | NI | CODEX/ADO/BODEX/DOSE |
| Hu et al.(2015) | NI | NI | CURB-65/BAP-65/PSI |
| Echevarria et al.(2016) | Hosmer–Lemeshow | multiple imputation | DECAF/CURB-65/CAPS/APACHEⅡ/BAP-65 |
| Gayaf et al.(2021) | NI | NI | BAP-65/CURB-65/DECAF/PSI |
| Telukutla et al.(2020) | sensitivity, specificity, PPV, NPV and accuracy | NI | DECAF/BAP-65 |
| Zidan et al.(2020) | sensitivity, specificity | NI | DECAF/ modified DECAF |
| Echevarria et al.(2019) | Hosmer–Lemeshow | multiple imputation | NEWS/NEWS2 |
| Almagro et al.(2019) | Hosmer–Lemeshow | NI | CODEX/mCODEX |
| Emergency department | | |  |
| Hodgson et al.(2017) | Hosmer–Lemeshow、 Calibration plots | there were no missing data | NEWS/CREWS/ salford-NEWS |
| Germini et al.(2019) | sensitivity、specificity | multiple imputations | BAP-65 |
| ICU | | |  |
| Shi et al.(2019) | NI | NI | DECAF/ v-DECAF/CURB-65/GCS/APACHEⅡ/BAP-65 |

Table S9: Data from 15 studies used for meta-analysis

| **Study** | **Time** | **Name** | **AUC** | **95%CI** |
| --- | --- | --- | --- | --- |
| Almagro et al.(2014) | 90-day mortality | CODEX | 0.72 | 0.69-0.75 |
| Almagro et al.(2014) | 90-day mortality | ADO | 0.65 | 0.58-0.65 |
| Almagro et al.(2014) | 90-day mortality | BODEX | 0.62 | 0.58-0.65 |
| Almagro et al.(2014) | 90-day mortality | DOSE | 0.6 | 0.57-0.63 |
| Almagro et al.(2014) | 1-year mortality | CODEX | 0.68 | 0.65-0.71 |
| Almagro et al.(2014) | 1-year mortality | ADO | 0.64 | 0.61-0.67 |
| Almagro et al.(2014) | 1-year mortality | BODEX | 0.51 | 0.48-0.55 |
| Almagro et al.(2014) | 1-year mortality | DOSE | 0.59 | 0.56-0.63 |
| Hu et al.(2015) | In-hospital mortality | CURB65 | 0.744 | 0.680-0.809 |
| Hu et al.(2015) | In-hospital mortality | BAP-65 | 0.665 | 0.594-0.736 |
| Hu et al.(2015) | In-hospital mortality | PSI | 0.847 | 0.799-0.895 |
| Echevarria et al.(2016) | In-hospital mortality | DECAF | 0.82 | 0.79-0.85 |
| Echevarria et al.(2016) | In-hospital mortality | CURB65 | 0.76 | 0.72-0.80 |
| Echevarria et al.(2016) | In-hospital mortality | CAPS | 0.77 | 0.73-0.81 |
| Echevarria et al.(2016) | In-hospital mortality | APACHEⅡ | 0.78 | 0.74-0.82 |
| Echevarria et al.(2016) | In-hospital mortality | BAP-65 | 0.77 | 0.73-0.81 |
| Echevarria et al.(2016) | 30-day mortality | DECAF | 0.79 | 0.75-0.83 |
| Echevarria et al.(2016) | 30-day mortality | CURB65 | 0.73 | 0.69-0.77 |
| Echevarria et al.(2016) | 30-day mortality | CAPS | 0.73 | 0.69-0.77 |
| Echevarria et al.(2016) | 30-day mortality | APACHEⅡ | 0.72 | 0.68-0.77 |
| Echevarria et al.(2016) | 30-day mortality | BAP-65 | 0.72 | 0.68-0.76 |
| Hodgson et al.(2017) | In-hospital mortality | NEWS | 0.74 | 0.66-0.82 |
| Hodgson et al.(2017) | In-hospital mortality | CREWS | 0.72 | 0.63-0.80 |
| Hodgson et al.(2017) | In-hospital mortality | salford-NEWS | 0.62 | 0.53-0.70 |
| Almagro et al.(2019) | 90-day mortality | CODEX | 0.653 | 0.55-0.755 |
| Almagro et al.(2019) | 6-month mortality | CODEX | 0.678 | 0.6-0.752 |
| Almagro et al.(2019) | 1-year mortality | CODEX | 0.658 | 0.604-0.712 |
| Almagro et al.(2019) | 5-year mortality | CODEX | 0.691 | 0.644-0.737 |
| Almagro et al.(2019) | 10-year mortality | CODEX | 0.737 | 0.675-0.779 |
| Almagro et al.(2019) | 90-day mortality | mCODEX | 0.664 | 0.566-0.759 |
| Almagro et al.(2019) | 6-month mortality | mCODEX | 0.684 | 0.61-0.757 |
| Almagro et al.(2019) | 1-year mortality | mCODEX | 0.66 | 0.606-0.713 |
| Almagro et al.(2019) | 5-year mortality | mCODEX | 0.693 | 0.647-0.737 |
| Almagro et al.(2019) | 10-year mortality | mCODEX | 0.729 | 0.668-0.771 |
| Germini et al.(2019) | In-hospital mortality | BAP-65 | 0.64 | 0.59–0.68 |
| Gayaf et al.(2021) | 30-day mortality | BAP-65 | 0.691 | 0.608-0.766 |
| Gayaf et al.(2021) | 30-day mortality | CURB65 | 0.77 | 0.691-0.836 |
| Gayaf et al.(2021) | 30-day mortality | DECAF | 0.663 | 0.579-0.740 |
| Gayaf et al.(2021) | 30-day mortality | PSI | 0.712 | 0.630-0.785 |
| Gayaf et al.(2021) | 90-day mortality | BAP-65 | 0.672 | 0.588-0.749 |
| Gayaf et al.(2021) | 90-day mortality | CURB65 | 0.715 | 0.633-0.788 |
| Gayaf et al.(2021) | 90-day mortality | DECAF | 0.743 | 0.663-0.813 |
| Gayaf et al.(2021) | 90-day mortality | PSI | 0.686 | 0.602-0.761 |
| Telukutla et al.(2020) | In-hospital mortality | DECAF | 0.965 | 0.936-0.993 |
| Telukutla et al.(2020) | In-hospital mortality | BAP-65 | 0.712 | 0.6-0.823 |
| Zidan et al.(2020) | In-hospital mortality | DECAF | 0.92 | 0.862-0.978 |
| Zidan et al.(2020) | In-hospital mortality | modified DECAF | 0.948 | 0.905-0.991 |
| Echevarria et al.(2019) | In-hospital mortality | NEWS | 0.65 | 0.61-0.68 |
| Echevarria et al.(2019) | In-hospital mortality | NEWS2 | 0.7 | 0.67-0.74 |
| Echevarria et al.(2019) | In-hospital mortality | NEWS2 all copd | 0.72 | 0.68-0.76 |
| Shi et al.(2019) | 90-day mortality | APACHEⅡ | 0.848 | 0.776-0.919 |
| Shi et al.(2019) | 90-day mortality | CURB65 | 0.606 | 0.489-0.723 |
| Shi et al.(2019) | 90-day mortality | GCS | 0.622 | 0.510-0.733 |
| Shi et al.(2019) | 90-day mortality | BAP-65 | 0.617 | 0.506-0.727 |
| Shi et al.(2019) | 90-day mortality | DECAF | 0.777 | 0.676-0.878 |
| Shi et al.(2019) | 90-day mortality | v-DECAF | 0.852 | 0.766-0.938 |
| Shiroshita et al.(2022) | in-hospital mortality | BAP-65 | 0.69 | 0.66-0.72 |
| Shiroshita et al.(2022) | in-hospital mortality | CURB65 | 0.69 | 0.66-0.72 |
| Mekanimitdee et al.(2021) | In-hospital mortality | CURB65 | 0.71 | 0.66-0.76 |
| Chen et al.(2021) | In-hospital mortality | BAP-65 | 0.732 | 0.693-0.768 |
| Chen et al.(2021) | In-hospital mortality | CRB65 | 0.734 | 0.695-0.770 |
| Chen et al.(2021) | In-hospital mortality | CURB65 | 0.787 | 0.751-0.82 |
| Chen et al.(2021) | In-hospital mortality | NEWS | 0.771 | 0.734-0.805 |
| Yu et al.(2020) | In-hospital mortality | CURB65 | 0.704 | 0.594-0.813 |
| Yu et al.(2020) | In-hospital mortality | DECAF | 0.923 | 0.872-0.974 |
| Yu et al.(2020) | In-hospital mortality | BAP-65 | 0.747 | 0.643-0.851 |
